# Driving Stress-Induced Effects on the Orofacial Region and Its Functions and Health Behaviors in Riyadh: A Cross-Sectional Survey

**DOI:** 10.3390/healthcare12151538

**Published:** 2024-08-02

**Authors:** Cristalle Soman, Aya Tarek Faisal, Malak Mohamed Alsaeygh, Abdulrahman Dahham Al Saffan, Ra’ed Ghaleb Salma

**Affiliations:** 1 Department of Oral Maxillofacial Surgery and Diagnostic Sciences, College of Medicine and Dentistry, Riyadh Elm University, Riyadh 11681, Saudi Arabia; 2 College of Medicine and Dentistry, Riyadh Elm University, Riyadh 11681, Saudi Arabia; 3 Dental Public Health, Ministry of Health, Qassim Health Cluster, Qassim 13225, Saudi Arabia

**Keywords:** driving, stress, health behavior, stress response, habits, masticatory muscles, myofascial pain, oral parafunction, bruxism, pain, temporomandibular disorders

## Abstract

Driving stress is a multifaceted phenomenon, and the experience of driving invokes stress. Driving causes the activation of stress-response mechanisms, leading to short-term and long-term stress responses resulting in physiological and behavioral changes. The aim of this study was to evaluate driving stress-initiated effects on orofacial functions and health behaviors in the Riyadh population. A cross-sectional survey was conducted in Riyadh using a pre-validated set of questionnaires for habitual information, a driving stress assessment using a driving-behavior inventory, and an assessment of parafunctional habits and effects on orofacial functions. The results indicate that nearly 50% of the sample spends more than two hours commuting, and more than 50% of the sample has inadequate sleep and insufficient exercise. Oral parafunctional habits like nail biting (*p* = 0.039) and lip or object biting (*p* = 0.029) had a significant correlation with aggressive driving behaviors, whereas the grinding of teeth (*p* = 0.011), the clenching of jaws (*p* = 0.048), lip or object biting (*p* = 0.018), and pain in mastication (*p* = 0.036) had a positive correlation with driving dislikes. Driving stress can be detrimental to one’s health and not only impacts health behaviors but also induces oral parafunctional habits and adversely affects orofacial regions and functions. Acute driving stress responses may be transient. However, prolonged driving stress can be maladaptive and can increase the risk of chronic diseases including chronic temporomandibular joint disorders and parafunctional habit-related changes in the oral cavity.

## 1. Introduction

Driving experience invokes stress, which harms one’s overall health [1]. Nowadays, much driving time is spent commuting to workplaces, universities, hospitals, schools, and other destinations [2]. Driving stress is a multifaceted phenomenon defined as confronting a circumstance where the apparent demand, which is generally created based on earlier experiences of driving, inner body emotional states, and outside stimuli, is greater than the existing resources [3,4]. Driving stress may also be associated with traffic factors and can lead to road traffic accidents or incidents (RTAs or RTIs). 

Saudi Arabia, in the last decade, was reported to have one of the highest mortality and morbidity rates due to road traffic accidents or incidents (RTAs or RTIs) in the Eastern Mediterranean region of the world, as reported by the Global Status Report on Road Safety of the World Health Organization (WHO) in 2015 [5,6]. RTA-associated mortality was also reported to be in the leading front in Saudi Arabia during 1990–2010, as estimated by the Global Burden of Diseases 2010 (GBDs 2010), which evaluated the country-level burden of diseases in KSA. The same report disclosed 11.75% of the entire mortality in 2010. An age range of 15 through 54 years was found to have the highest concentration of RTA injuries [7]. RTA-associated fatalities were on the rise over the past decade, from 17.4 to 24 per 100,000 of the population in comparison to the USA (10) and the UK (5), where appropriate road safety and preventive measures have been implemented [6,8]. Among the high-income countries, Saudi Arabia topped the deaths arising from RTAs, with an accident-to-death ratio of 32:1. An RTA is considered to be the foremost reason of death for males in the age group of 16 to 30 years old. Saudi Arabia also stood out in the accident-to-injury ratio (8:6) compared to the international ratio, which was reported to be 8:1.8, with most RTAs worldwide being caused by four wheelers. The country recorded 86,000 deaths and 611,000 injuries, resulting in 7% of permanent disabilities being due to RTAs [8]. Riyadh, the most crowded city in Saudi Arabia, has reported more RTIs. Heavy traffic, unusual weather patterns with rains and sandstorms, and a high inflow of population density during Hajj can also contribute to RTAs [5].

In the above context, during 2016, Saudi Arabia laid down the National Transformation Plan (NTP) in which road safety was one of the pillars of reforms. At that time (2016), Saudi Arabia had a fatality rate due to RTAs of 28.8/100,000 population. The National Ministerial Traffic Safety Committee (NMTSC) was established in 2018 to address the concern for road safety and traffic accidents. With the support of the WHO towards the road safety vision, the RTA rates dropped by 2021 to 18.5 per 100,000 population. The country envisages reducing RTAs to 6–10 per 100,000 by 2030 [8,9,10]. Many strategies have been successfully employed to reduce RTA rates. It was observed that there were three main factors associated with the risk and severity of RTA-associated mobile phone usage while driving, namely, over speeding, breaking traffic rules like not wearing seat belts, etc.

Nevertheless, many countries have introduced a green mode of transport using two- or three-wheeled micro-mobility e-scooters, including Saudi Arabia, to combat high traffic congestion. However, there are a number of challenges such as climatic, cultural, operational, safety, and economical concerns in the use of e-scooters or scooter sharing in Saudi Arabia, which include extreme temperature rises in the summer, cultural blockages towards public transport usage, infrastructure sufficiency to support e-scooters, and dedicated traffic lanes [11]. The usage of e-scooters also poses a risk to traumatic injuries and is an emerging phenomenon [12]. A systematic review conducted on the safety of e-scooters found that there was a great necessity for analyzing e-scooter interactions with other road users. The data revealed that the most common injuries susceptible among e-scooter users were to the head and face during collisions [13].

Nowadays, research, therefore, considers driving stress, irrespective of any mode of driving on the road, as a health-related behavior [2]. A variety of factors influence the number of RTAs and their severity. Some of these factors include the characteristics of (a) the individual driving the vehicle, such as their age and gender; (b) the vehicle, including its type, height, tire condition, and age; (c) the roadway, including the number of lanes, lane and shoulder widths, intersections, and roadside conditions; and (d) crashes, including exceeding speed limits, not wearing seatbelts, the usage of a phone, breaking traffic laws, etc. [8,14]. Interestingly, particular objects on the road, such as cyclists, heavy vehicles like trucks, and infrastructures like intersections, are highly associated with higher subjective stress levels. The effects of traffic conditions, such as high and low traffic, and the regions of driving, such as highways or cities, also influence driving stress [15,16,17]. The distance to be followed in the car, the distance from the lead vehicle, and behaviors could also affect drivers’ psychological states, such as emotions, initiations of stress, and workloads [4]. Driving vehicles for long distances or for hours at a time can also cause an increase in stress levels that extend over a certain amount of time, even after the event of driving itself [1]. Long-distance drives can compromise the health of individuals, including stress caused by traffic congestion, parking space searches, driver-to-driver interactions, and safety issues, collectively termed travel impedance. The extended time in long driving distances also affects other factors, leading to a lack of sufficient sleep; a reduction in family time; inadequate leisure time, physical activity, or cooking time; and sedentary sitting, which is linked to an increased risk of chronic conditions. Cross-sectional research from the United States of America has also revealed that the time spent per hour in a car was associated with the risk of developing obesity by 6% [2]. Thus, driving stress, within its driving domain, can be related to state stress and trait stress, defined as stress relating to definite external circumstances or incidents and stress arising from within the individual, respectively [1].

In a recent study, multimodal realistic statistics were used to understand the correlation between stress-level changes in drivers and emotions while on-road driving. Various road objects were associated with different elements of negative facial emotions, which correlated with different stress levels [4]. The cognitive-emotional standing of a driver may be identified by analyzing facial expressions, as a variety of emotions can be activated by facial muscles [18]. These studies highlight the involvement of the orofacial structure and functions during stress induced by driving [4,18].

Stress elicits neuromuscular activity in the orofacial region and persuades maladaptive behavioral habits and parafunctional habits [19,20]. Stress and oral habits are also potential contributors to the etiology of painful TMDs [21,22,23,24,25]. The overall prevalence of TMDs among the general population was reported to be 34%. A recent meta-analysis, in 2024, reported the global prevalence of temporomandibular disorders to be 34%, with the age range of 18–60 years being analyzed as having TMDs [26,27]. Parafunctional oral habits like clenching, bruxism, indirectly causes the initiation of temporomandibular joint disorders (TMDs), orofacial pain (myofascial pain), and headaches [15,19,20,21]. A longitudinal study was conducted with a multilevel model to evaluate the association of emotional distress, tension in the muscles of mastication, and pain in temporomandibular joint disorders (TMDs). The study found masticatory muscle tension to be a causal factor in TMD pain [20].

Oral habits such as bruxism during wakefulness are influenced by stress [19,20]. Thus, bruxism during driving is awake bruxism. Awake bruxism is defined as an awareness of the clenching of the jaws or as an activity of the masticatory muscles during wakefulness [21]. It is characterized by repeated or sustained tooth contact with a thrusting of the lower jaw bone [22]. Awake bruxism is reported to be present among 20% of the adult population [21]. A recent systematic review on adult awake bruxism showed an overall pooled prevalence of 15.44%, with no differentiation being found between different genders. Awake bruxism may worsen discomfort and strain in the stomatognathic orofacial structures [19]. Stress has been considered to be the most common factor associated with bruxism. Stress leads to bruxism, which can then increase stress hormone levels, making bruxism more likely to occur again in a self-strengthening loop [23,24]. The primary tool in the assessment of bruxism in clinical practice and research is self-reported assessments. These self-assessment tools are useful in evaluating stress and anxiety as well as joint and muscle pains. These assessments thus help to understand the association of stress and its effect on joint and muscular inflammation, resulting in pain and impaired functions [25].

Individuals also succumb to deleterious health habits while stressed which affect oral health, like smoking and an excessive consumption of coffee and carbonated soft drinks to manage fatigue. Saudi Arabia, being a high-income country and with a boom in economic growth, has also garnered variations in health and lifestyle behaviors. The country has a low prevalence of physical activity that is exacerbated by the hot weather conditions, increased urbanization, and poor diet patterns that slacken the promotion of one’s physical health. The prevalence of smoking among females amounts to 1.4% in 15- to 64-year-olds and hikes to about 24.2% among 15- to 64-year-old males, and clearly, males are more likely to smoke than females [7]. According to a World Health Organization report on road traffic injuries (2023), there are many reasons for impaired driving due to distractions [14]. A higher proportion of road traffic injuries, reaching up to two thirds, was reported to occur among working people in the age group of 18 to 59 years. Males are more often likely to face fatal injuries in comparison to females. An increase in the speed of a car is directly proportional to the risk of a crash and the extent of the severity of the crash and injuries [7,14].

Driving stress can also increase the risk of RTAs. Given the above background, evaluating the correlation of driving stress with orofacial functions and health habits is inevitable, which explains the rationale for this study. Understanding these associations will benefit mitigating the burden of driving stress on health behaviors and orofacial functions based on the degree of its impact. Thus, this study aimed to evaluate the driving stress-initiated effects of driving stress on the orofacial functions and health behaviors in the Riyadh population.

The null hypothesis postulated for this study was as follows: ‘There is no significant difference in orofacial health, its functions, and health behaviors between individuals who experience high levels of driving stress and those who experience low levels of driving stress in the Riyadh population’. The alternate hypothesis was as follows: ‘There is a significant difference in orofacial health, its functions, and health behaviors between individuals who experience high levels of driving stress compared to those who experience low levels of driving stress in the Riyadh population’.

## 2. Materials and Methods

Study design: A cross-sectional study was conducted using a randomized sampling technique after ethical clearance from the university.

Data collection methods: Description of the questionnaire: this study was conducted using self-reported pre-validated questionnaires, with three sections from previous studies by Ding et al., 2014, Chung et al., 2019, Almutairi et al., 2021, and Atsü et al., 2019 [2,28,29,30].

Section 1: Demographic and habitual information were collected, such as age, gender, marital status, education, employment status, region of residence, hours spent driving in a day, smoking habits, consumption of beverages, excessive sitting, insufficiently active, insufficient sleep, time stress, and social functioning [2].

Section 2: The second division focuses on assessing driving stress based on driving behaviors using the self-reported Driver Behavior Inventory (DBI), which is based on five factors, namely, aggression during driving; aversion to driving; tension; and frustration linked to overtaking, annoyance when being overtaken, and sharp attentiveness and concentration, which were evaluated on a 4-point Likert scale ranging from 0 to 4 [28].

Section 3: This section focuses on the effects of driving stress on the orofacial region and functions, which were assessed by (a) parafunctional habits during driving stress, such as a biting of the nails, grinding of the teeth, clenching of the jaws, lip or object biting, and chewing gum, with all parameters being rated on a 4-point Likert scale from 0 to 4 [29], and (b) temporomandibular joint disorder (TMD) symptoms, including pain in the facial region, any episodes of a headache, the audibility of TMJ sounds, a limitation in the opening of the oral cavity and its closure, pain in mastication, and tiredness in the muscles, which were recorded in a dichotomous yes/no format [30]. Since the study design was self-reported by the population, a standard temporomandibular disorder pain-screening questionnaire used by clinicians was used. For simplicity in understanding for the general population while self-reporting, a yes/no format was chosen for TMD evaluation.

Study population: The Riyadh population was the target sample. Participants were invited to participate in this research voluntarily, and after obtaining digital informed consent, created online, an invitation to participate using an electronic questionnaire designed in Google Forms was distributed to the public using social media platforms from March 2024 to April 2024, with the following eligibility criteria.

Inclusion criteria: a person fulfilling all the listed criteria below was included in this study:

- Age 18 years to 78 years;
- Has a valid driving license;
- Drives primarily in Riyadh.

Exclusion criteria: a person who had encountered any of the following conditions in their lifetime was excluded from this study:

- A previously diagnosed neuromuscular disorder;
- An accident/injury to the neck;
- A history of head and neck surgeries;
- Developmental and congenital diseases in the head and neck.

Sampling technique: Simple random sampling was used. According to the Raosoft sample size calculator, the minimum sample size needed for this study, with a 95% confidence level, an 8% margin of error, and a 50% response distribution, to represent the Riyadh population was 151. The above parameters of an 8% margin of error and a 50% response rate were calculated and acceptable, as this was a preliminary exploratory study. The representative sample was collected following the aforementioned eligibility criteria.

Modes of questionnaire administration: An online self-administered survey with a questionnaire was created using Google Forms and was distributed in and outside the university, classrooms, and social media for various dental universities in Saudi Arabia.

Sample recruitment: Simple random sampling was used to recruit the study participants. A participant who was voluntarily willing to participate in this study proceeded to complete the questionnaire.

Information on the entry process: A brief outline of this study was provided, and voluntary participants were invited to participate in this research via an online invitation. To avoid duplications, responses were limited to a single response.

Study preparation: a training session was conducted for all investigators for the orientation, questionnaire, and advertising.

Statistical analysis: The data collected were analyzed using the Statistical Package for the Social Sciences (SPSS, version 26). Descriptive statistics were used for categorical and continuous data for demographic and habitual characteristics. A correlation analysis was performed to associate driving stress and its effects on the orofacial structure. A value of *p* less than 0.05 was considered significant (with the assumption that there is no effect or difference between the groups). Binary logistic regression and a bivariate analysis evaluated the association between driving stress, driving behaviors, and effects on the orofacial region and functions.

## 3. Results

The present study investigated subjects who were experiencing driving stress. Their demographic data include personal details like age, gender, education, marital status, employment status, place of residence, and driving hours. In this study, 161 subjects with driving stress were assessed and included. The demographic data (Table 1) show that the age of the subjects ranged from 18 to 58 years, with 55.9% of the subjects being of the age 18–28 years, 33.5% being between 29 and 38 years, 8.7% being between 39 and 48 years, and 1.9% being between 49 and 58 years. A total of 53.4% of the subjects in this study were males, and the remaining were females. A total of 71.4% of the subjects in this study were single, whereas 28.6% of them were married. A total of 51.6% of these subjects had a bachelor’s degree, whereas 26.1% had a high school/diploma. A total of 65.2% of the subjects in this study were employed, whereas the remaining were either students or unemployed. A total of 64.0% of the subjects lived in the city. Almost 49.1% of participants spent more than 2 h driving.

Table 2 describes the habitual information of the subjects, where 29.8% of the subjects in this study smoked while driving. Interestingly, 68.9% of the subjects diagnosed with driving stress consume beverages while driving. A majority of the subjects, i.e., 54.0% of them, drink coffee while driving, whereas 11.8% of them consume tea. A total of 65.2% of the subjects in this study who experience driving stress spend more than 8 h sitting, which is excessive. Similarly, 65.2% have insufficient physical activity. A total of 66.5% of the subjects in this study who experience driving stress have insufficient sleep, i.e., less than 7 h per day. Time stress was always observed among 36% of the subjects, whereas it was often observed among 22.4% of them. A total of 34.8% of the participants in this study had health problems interfering with social activities.

**Figure 1:**
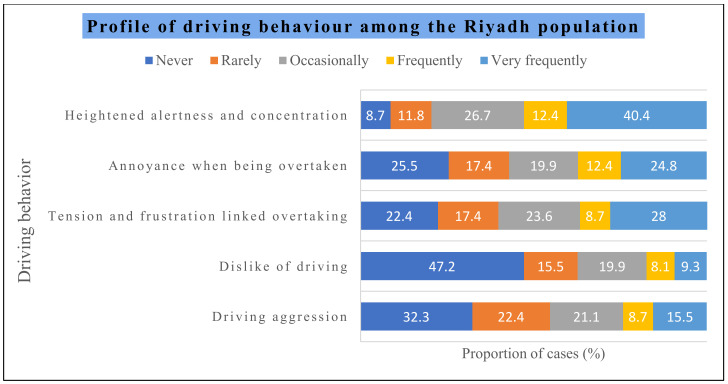
Profile of driving behaviors among the subjects.

The data in Figure 1 depict driving behaviors among the Riyadh population with driving stress. Driving aggression was never observed among 32.3% of the subjects, rarely among 22.4% of the subjects, occasionally among 21.1% of the subjects, frequently among 8.7% of the subjects, and very frequently among 15.5% of the subjects. Similarly, a dislike for driving was not observed among 47.2% of the subjects, rarely among 15.5% of the subjects, occasionally among 19.9% of the subjects, frequently among 8.1% of the subjects, and very frequently among 9.3% of the subjects. Tension and frustration linked to overtaking were not present among 22.4% of the subjects, rarely among 17.4% of the subjects, occasionally among 23.6% of the subjects, frequently among 8.7% of the subjects, and very frequently among 28.0% of the subjects. Annoyance when being overtaken was not evident among 25.5% of the subjects, rarely among 17.4% of the subjects, occasionally among 19.9% of the subjects, frequently among 12.4% of the subjects, and very frequently among 24.8% of the subjects. Heightened alertness and concentration were never observed among 8.7% of the subjects, rarely among 11.8% of the subjects, occasionally among 26.7% of the subjects, frequently among 12.4% of the subjects, and very frequently among 40.4% of the subjects.

**Figure 2:**
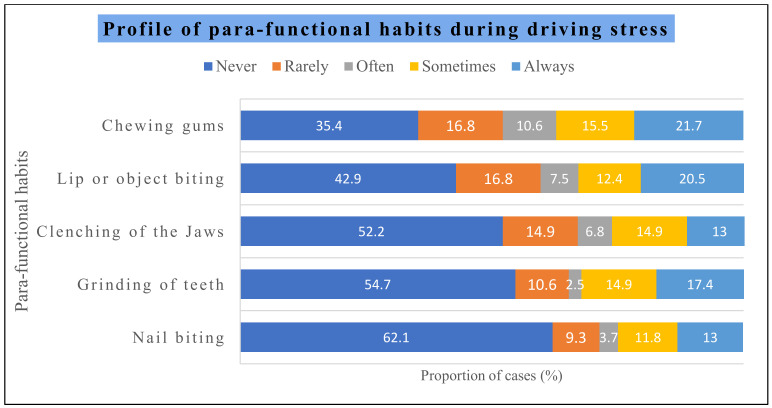
Profile of parafunctional habits during driving stress.

The association of parafunctional habits during driving stress, as detailed in Figure 2, showed that nail biting was not a significant habit among 62.1% of the subjects. It was rarely reported among 9.8% of the subjects, often among 3.7% of the subjects, sometimes among 11.8% of the subjects, and always among 13.0% of the subjects.

The grinding of teeth was never observed among 54.7% of the subjects, rarely among 10.6% of the subjects, often among 2.5% of the subjects, sometimes among 14.9% of subjects, and always among 17.4% of the subjects. A clenching of the jaws was never observed among 52.2% of the subjects, rarely among 14.9% of the subjects, often among 6.8% of the subjects, sometimes among 14.9% of the subjects, and always among 13.0% of the subjects. Lip or object biting was never observed among 42.9% of the subjects, rarely among 16.8% of the subjects, often among 7.5% of the subjects, sometimes among 12.4% of the subjects, and always among 20.5% of the subjects. Chewing gum was never observed among 34.4% of the subjects, rarely among 16.8% of the subjects, often among 10.6% of the subjects, sometimes among 15.5% of the subjects, and always among 21.7% of the subjects.

**Figure 3:**
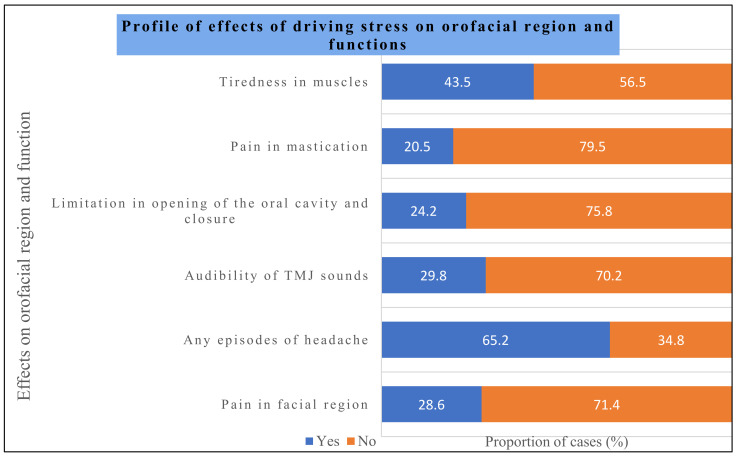
Profile of effects of driving stress on orofacial region and functions.

Figure 3 provides insights into the effects of driving stress on the orofacial region and functions. A total of 28.6% of the subjects had pain in the facial region, 65.2% of the subjects in this study had episodes of a headache, 29.8% of the subjects had an audibility of TMJ sounds, 24.2% of the subjects had limitations in opening and closing the oral cavity, 20.5% of the subjects had pain in mastication, and 43.5% of the subjects had tiredness in their muscles.

A correlation analysis of the data from the present study, whose results are shown in Table 3, revealed that nail biting and driving aggression showed a positive significant correlation, i.e., driving aggression affected the parafunctional stress habit of nail biting when driving under stress. The grinding of teeth and driving dislikes showed a positive significant correlation, i.e., driving dislikes affected the parafunctional stress habit of grinding teeth when driving under stress. A clenching of the jaws and driving dislikes showed a significant positive correlation, i.e., driving dislikes affected the parafunctional stress habit of the clenching of jaws when driving under stress. Lip or object biting and driving aggression showed a positive significant correlation, i.e., driving aggression affected the parafunctional stress habit of lip or object biting when driving under stress. Similarly, lip or object biting and driving dislikes showed a significant positive correlation, i.e., driving dislikes affected the parafunctional stress habit of lip or object biting when driving under stress. Chewing gum and heightened alertness and concentration showed a significant positive correlation, i.e., chewing gum was associated with heightened alertness and concentration when driving under stress.

Pain in the facial region and annoyance, when overtaken, showed a significant positive correlation, i.e., annoyance affected the orofacial structure and functioning that caused pain in the facial region. An audibility of TMJ sounds and annoyance, when overtaken, showed a positive significant correlation, i.e., annoyance affected the orofacial structure and functioning that caused the audibility of TMJ sounds among the subjects.

Limitations in the opening of the oral cavity and closure and annoyance when being overtaken showed a positive significant correlation, i.e., annoyance had an effect on the orofacial structure and functioning that caused limitations in the opening and closure of the oral cavity among the subjects. Pain in mastication and annoyance, when overtaken, showed a significant positive correlation, i.e., annoyance affected the orofacial structure and functioning that caused pain in mastication and chewing among the subjects.

Similarly, pain in mastication and driving dislikes showed a significant positive correlation, i.e., a dislike of driving was associated with the orofacial structure and functioning, which caused pain in mastication and chewing among the subjects.

The results of the association between driving behaviors and their effects on the orofacial region and functions are detailed in Table 4. The R^2^ values indicate a goodness of fit of the study data on driving behaviors and their effects on the orofacial functions to the regression model. The value of R^2^ ranges from 0 to 1, where an A value closer to 1 suggests that driving behaviors account for a larger portion of the variation in orofacial effects, meaning that it is a stronger predictor. A value closer to 0 indicates that driving behaviors account for very little of the variation, suggesting that other factors might be more influential. Other driving behaviors showed no significant likelihood of factors affecting pain in the facial region. None of the driving behaviors showed a significant effect on the cause of episodes of headaches among the subjects. Driving behaviors, like annoyance when being overtaken, were significantly more likely the factor responsible for the audibility of TMJ sounds. Other driving behaviors showed no significant likelihood of factors affecting the audibility of TMJ sounds.

Similarly, driving behaviors like annoyance when overtaken were significantly more likely the factor responsible for limitations in the opening and closure of the oral cavity. Other driving behaviors did not show any significant likelihood of factors affecting limitations in the opening and closure of the oral cavity. Driving behaviors like a dislike of driving and annoyance when overtaken had a significant influence and were more likely the factors responsible for pain in mastication and chewing. Other driving behaviors showed no significant likelihood of factors affecting pain in mastication and chewing. None of the driving behaviors significantly affected the cause of muscle tiredness among the subjects.

Also, driving behaviors like annoyance when being overtaken were significantly more likely (β = 1.46, 95% CI = 1.15–1.86, *p* = 0.002) the factor responsible for pain in the facial region, and annoyance when being overtaken was significantly more likely (β = 1.43, 95% CI = 1.13–1.81, *p* = 0.003) the factor responsible for the audibility of TMJ sounds. Similarly, annoyance when being overtaken was significantly more likely (β = 1.28, 95% CI = 1.00–1.63, *p* = 0.049) the factor responsible for limitations in the opening and closure of the oral cavity. A dislike of driving and annoyance when being overtaken had significant influence and were more likely (β = 1.33, 95% CI = 1.02–1.75, *p* = 0.039) the factors responsible for pain in mastication and chewing.

Table 3 and Table 4 illustrate the individual and independent associations between orofacial health, its function, and health behaviors. The data analysis for this study concluded that nail biting and driving aggression showed a positive significant correlation (r = 0.163, *p* = 0.039). The grinding of teeth and driving dislikes showed a positive significant correlation (r = 0.201, *p* = 0.011). A clenching of the jaws and driving dislikes showed a positive significant correlation (r = 0.156, *p* = 0.048). Lip or object biting and driving aggression showed a positive significant correlation (r = 0.173, *p* = 0.029). Lip or object biting and driving dislikes showed a positive significant correlation (r = 0.187, *p* = 0.018). Chewing gum and heightened alertness and concentration showed a positive significant correlation (r = 0.162, *p* = 0.041). Pain in the facial region and annoyance when being overtaken showed a positive significant correlation (r = 0.252, *p* = 0.001). The audibility of TMJ sounds and annoyance when being overtaken showed a positive significant correlation (r = 0.241, *p* = 0.002). Limitations in the opening and closure of the oral cavity and annoyance when being overtaken showed positive significant correlation (r = 0.157, *p* = 0.047).

**Figure 4:**
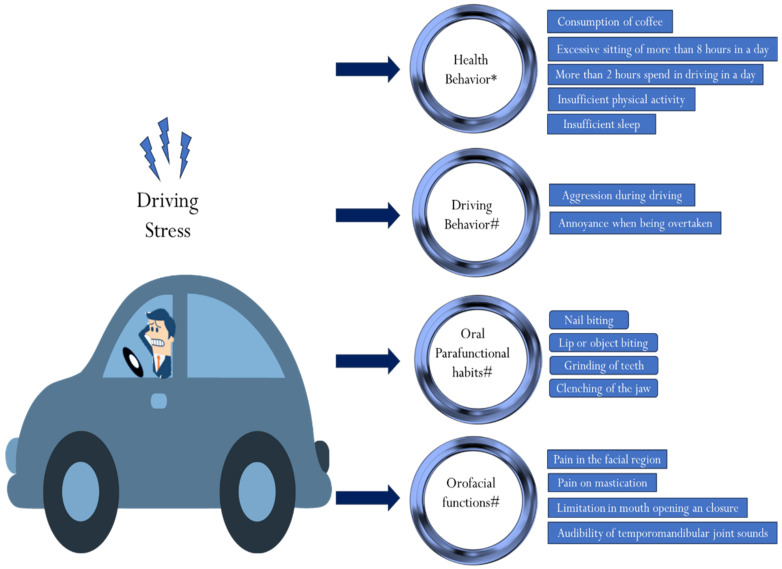
Summary of the results, indicating that driving stress has detrimental effects on health behaviors, driving behaviors, and orofacial functions and induces parafunctional habits. * indicates the results are prevalent in more than 50% of the population; # indicates the parameters have statistically significant correlations to driving stress (r = 0.157, *p* = 0.047). Pain in mastication and annoyance when being overtaken showed a positive significant correlation (r = 0.166, *p* = 0.036). Pain in mastication and driving dislikes showed a positive significant correlation (r = 0.244, *p* = 0.002).

Therefore, at least one primary event is significantly associated with predicting differences in orofacial health and functions and health behaviors between individuals who experience high levels of driving stress compared to those who experience low levels of driving stress (Figure 4), which results in rejecting the null hypothesis.

The *p* value in this study for the correlation analysis was evaluated as less than 0.05 being statistically significant. Due to this, the probability of driving stress to be linked to orofacial health, its functions, and health behaviors by a random chance was low (less than 5%). This suggests that this study accumulated adequate evidence to reject the null hypothesis and support the alternative hypothesis, where there was likely a real effect or relationship between driving stress and orofacial health, its functions, and health behaviors.

## 4. Discussion

Driving stress is a multifaceted phenomenon that may invoke anxiety, parafunctional habits, and health behaviors. The present study was conducted to evaluate the effects of driving stress on orofacial functions and health behaviors in Riyadh.

In this study, more than half of the participants had the following health behaviors: a habit of coffee consumption while driving, a commute of more than 2 h, excessive sitting of more than 8 h, inadequate sleep, and insufficient physical activity. Among the parafunctional habits, nail biting, lip or object biting, and driving aggression showed a positive significant correlation. The grinding of teeth, the clenching of jaws, lip or object biting, pain in mastication, and driving dislikes showed a positive significant correlation. Chewing gum was associated with heightened alertness and concentration when driving under stress. Annoyance when being overtaken affected the orofacial structure, causing limitations in the opening and closure of the oral cavity, pain in the facial region, and caused an audibility of TMJ sounds among the subjects. Annoyance, when overtaken, affected the orofacial structure and functioning and caused pain in mastication among the subjects.

**Figure 5:**
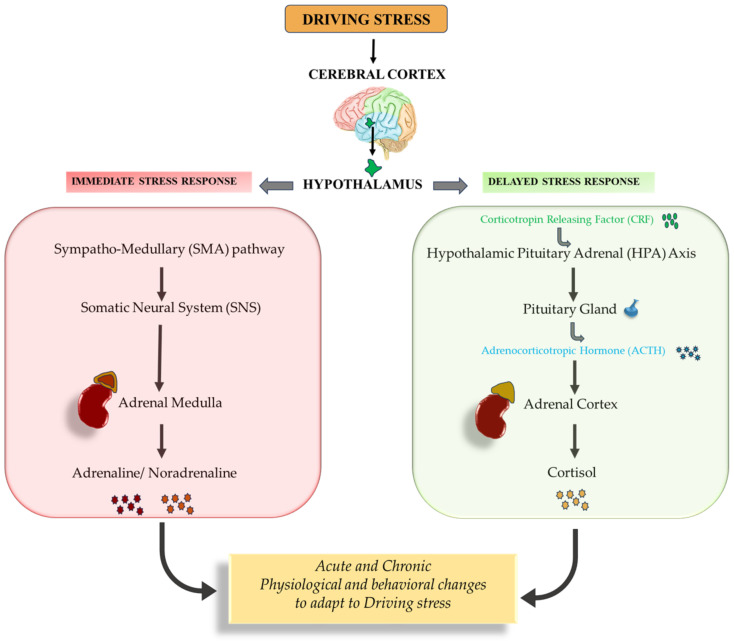
Driving stress-response mechanism.

Driving has an impact on health. Few mechanisms are proposed to explain the effects of driving on the health of an individual. One of the mechanisms of driving health impacts is due to prolonged hours of sitting, which is one of the sedentary behaviors posing health risk towards the development of cardiovascular and metabolic diseases. Another proposed mechanism is the similarity of physiological stress responses and driving-evoked acute stress. Acute stress during driving activates the cerebral cortex and hypothalamus. Two pathways of response are then activated (i) immediate response and (ii) delayed response (Figure 5). The immediate response is via the Sympatho-Medullary (SMA) pathway. This pathway is directly activated by the hypothalamus by evoking the Somatic Neural System (SNS) that stimulates the adrenal medulla to release adrenaline and noradrenaline. These hormones cause physiological changes in the body in preparation for an immediate response, such as a rise in blood pressure and heart rate. The delayed response to stress is enabled through the Hypothalamic Pituitary Adrenal (HPA) axis, mediated by the release of the corticotropin-releasing factor from the hypothalamus-releasing adrenocorticotropic hormone from the pituitary gland that stimulates the production and release of cortisol from the adrenal cortex [1]. The end products of both the pathways leads to physiological changes such as an increase in the heart rate; an increase in the blood pressure; an increase in the respiratory rate; an increase in the blood supply to the muscles; and an increase in muscular tension and behavioral changes, such as alertness and focused attention [31]. A decline in mental concentration is expected due to the inactivation of the SNS during the usage of driving distractors, such as mobile phones or navigators, which reduces the focus on driving. A stress response to driving circumstances such as traffic congestion increases cognitive demand and perceived stress while driving [1].

Previous studies evaluated various environmental disturbances and their influence on drivers’ stress levels, emotions, and anxiety [32,33]. Drivers’ emotional states are also influenced by conditions and situations in the cabin while driving, types of roads, drivers on the road, and the weather. These factors impact drivers’ performance and behaviors in manual and semi-automated driving [32]. Drivers’ heart rates were found to be associated with diverse appearances of the road, such as being shadowed by a vehicle, less distance from the lead vehicle in front while driving, and intersections, wherein an abrupt increase in the cardiac rate was noted, which might indicate the driver’s emotions and stress [33]. Drivers’ emotions and driving stress can also influence driving behaviors. Driving stress occurs when a need arises to confront a circumstance while driving where the demand is higher than the demand perceived by the driver. This perceived demand is accumulated from prior experiences while driving and internal and external bodily environments and stimuli [3]. Several studies attempted to evaluate this association between stress levels during driving and emotions by monitoring facial changes in expressions, temperatures recorded from the skin, conductance, and metrics to evaluate cardiac functions [34,35,36,37,38]. For instance, in a study, an increased heart rate correlated with increased negative emotions and driver stress levels [36]. Another study found that facial musculature movements were particular for each emotion and might be tracked to specific patterns [39].

From a psychological perspective, drivers’ emotions can be traced into the category of basic emotions. Basic emotions are distinct in their habitual fixed forms of physical and neural expressions, behavior patterns, and physiological markers like heart rates and movements including contractions of the facial musculature. Basic emotions can be elicited or become evident as a response to stimuli [31,40,41,42]. The above findings about facial muscular contractions are also reported in the present study, where parafunctional habits were found to have a significant correlation to driving dislikes, resulting in driving stress and anxiety. Our results support the findings of a previous study on the Saudi population which reported that parafunctional habits were prevalent in the Saudi adult population and were significantly associated with moderate-to-severe levels of anxiety [29].

Stress and anxiety significantly interplay with an individual’s health. Recent driving-related research studies have, therefore, shifted their focus on evaluating the impact of driving on health behaviors. A systematic review evaluated whether driving elicits an acute physiological stress response. The study found moderate evidence suggesting that driving for long hours elicits a stress response over an extended period [1]. The present study similarly reported that 49.1% of those participants who drove more than 2 h revealed the risk of driving stress development. Longer driving hours can also influence a driver’s health habits. Almost 49.1% of the participants spent more than 2 h driving, and 65.2% of the subjects in this study with driving stress had more than 8 h of sitting time during the day. More than half of the participants reported insufficient sleep and physical activity. A previous study examined the associations of driving time with a series of health behaviors using data from the Social, Economic, and Environmental Factor Study conducted in New South Wales, Australia, in 2010 and found that a longer driving time was associated with higher odds for smoking, insufficient physical activity, short sleep, obesity, and worse physical and mental health. The associations consistently showed a dose-response form, and more than 120 min of driving per day (12% of wake-up time in a day) had the most solid and constant associations with most health-behavior outcomes and affected adults that were middle-aged or older [2]. Extended driving hours also compromised time for other health behaviors, similar to the findings in our study. This, in turn, affected health habits such as inadequate sleep, a reduction in sleep hours [43,44], reduced social time with friends and family [45], and insufficient exercise or physical activities [45,46]. These results are comparable to the findings in our study. The findings of the present study are similar to these studies and buttress the finding of the impact of long driving hours being associated with deleterious health behaviors [1,2,43,44,45,46,47].

In the present study, the driver behavior inventory (DBI) was used to assess driving behaviors, and it was found that driving aggression was present frequently among 8.7% of subjects and very frequently among 15.5% of the subjects. Similarly, a dislike of driving was widespread among 9.3% of the subjects. Tension and frustration linked to overtaking were frequent among 28.0% of the subjects. Annoyance when being overtaken was frequent among 12.4% of subjects and very frequently among 24.8% of the subjects. Heightened alertness and concentration were predominantly frequent among 40.4% of the subjects. These findings support the stress response behaviors that are a result of driving stress [1,31].

Driving stress due to a dislike of driving was associated with a few TMJ disorder symptoms and parafunctions in this study. The results are similar to a previous study conducted in Turkey, in which oral parafunctions, especially bruxism and anxiety, were associated with the signs and symptoms of temporomandibular disorders in adolescents [30]. Another study evaluated stress and the orofacial area in terms of its influence of stress on oral habits, painful temporomandibular joint disorders, and health. The study highlighted that awake oral habits were influenced by stress, while there was no association of oral habits during sleep and stress [21]. A systematic review and meta-analysis investigated the association of stress and the oral habit of bruxism and found that stressed individuals had higher odds of bruxism than healthy individuals [22].

Stress can accelerate muscle toning and reduce one’s pain threshold. Recent evidence suggests that stress can initiate neurological degeneration, resulting in the diminution of critical highly implicated neural pathways in the involuntary muscle actions in the orofacial region. Bruxism can be worsened by the body’s stress response system. When stressed, a part of the brain called the hypothalamus triggers the release of hormones. These hormones cause the adrenal glands to produce corticosterone, a stress hormone. Cortisol, another stress hormone, is also elevated in the saliva during bruxism episodes. This hormonal response creates a loop [23].

In our study, driving stress due to a dislike of driving was associated with a few TMD symptoms and parafunctions. The results are similar to a study conducted in Turkey, in which oral parafunctions, especially bruxism and anxiety, were associated with temporomandibular disorders in adolescents [30]. Our results support the fact that TMDs arise due to multifactorial etiology. Oral parafunctions and emotions are contributing factors to TMDs. Driving stress causes emotional changes as found from the results of previous studies [34,35,36,37,38,39,40,41,42]. Hence, driving stress can indirectly be a causal factor of TMDs. No study has evaluated this correlation, except for the present study, which found a likely association between driving stress and TMDs.

The habit of chewing gum also activates the orofacial musculature and exerts an effect on the temporomandibular joints. The results of this study found a significant positive correlation among chewing gum and heightened alertness and concentration, which is consistent with previous findings [48]. This might be possibly due to the activation of the skeletal muscle and an increase in the blood supply to these muscles during the stage of acute stress response during driving. Parafunctional habits were found to have a significant correlation to driving dislikes, which causes driving stress and anxiety. A study on the Saudi population reported that self-reported parafunctional habits were prevalent in the Saudi adult population and significantly associated with moderate-to-severe levels of anxiety [29]. Hence, stress induced by driving can also exert a positive correlation to parafunctional habits.

The health effects of driving-induced stress are detrimental. Stress influences physiological and cognitive behavioral patterns. Hormones, neuroendocrine mediators, neurotransmitters, and peptides are released in response to stress. Chronic stress mediated by steroids may also cause variations in gene expressions and may alter cognitive expressions in the long term [49]. There is no sufficient scientific literature to support whether shorter driving distances can induce acute stress responses. It is noteworthy that stress responses to driving are related to certain situations of driving, such as traffic congestion, annoyance, and negative interactions with other drivers, etc., which increase cognitive demand and stresses perceived during the demand [1]. Driving stress is influenced not only by driving conditions but also by the drivers physical and behavioral changes. The initial stress levels and tiredness of the driver also have strong associations with stress, fatigue, and driving behaviors. Driving stress increases the risk of RTAs and chronic diseases in the long term [1,2,50]. Hence, adequate measures should be taken to lower the levels of driving stress.

The findings of this study highlight the impact of driving stress and its association with poor health-related behaviors and oral parafunctional habits associated with driving in the Riyadh population. This study also highlights that nearly 50% of the population in the city of Riyadh experience prolonged driving for more than 2 h, and more than 50% of the subjects reported insufficient sleep and exercise or activity. This study also found a positive correlation between parafunctional habits, temporomandibular joint disturbances, and driving stress. These factors point to the risk of chronic health disorders that can arise due to driving stress-related poor-health behaviors, such as obesity, cardiovascular disorders, TMJ pain, headaches, and orofacial pain. Hence, techniques to reduce driving stress should be considered while driving to prevent long-term health effects on an individual.

There are limitations to this study. Participants who needed help comprehending the English language had difficulty attempting the questionnaire and needed further translation of the facts into the local language. No clinical examination was employed in this study to verify the oral impact of parafunctions. The temporomandibular joint disorder questionnaire could not be employed, as it must be recorded by clinicians. This can be possible if the study in question is conducted in centers where patients belonging to the eligibility criteria can be examined and data can be recorded by the clinicians. Since this study is a preliminary exploratory study, the response rate of 50% and a margin of error set to 8% were acceptable. However, further studies should be conducted with an improved response rate and less margin of error. The sample size was adequate to represent Riyadh’s population, and, therefore, the results are generalizable. Future studies should focus on a regional translation of the questionnaire used for further studies. While the goodness of fit and power are related concepts in the sense that a better-fitting model is more likely to have higher power, they are distinct measures. The effect size of this study varied from a small-to-medium effect size (0.16 to 0.35) (Table 3 and Table 4). While effect size and power are important for confirmatory studies, they are less critical in exploratory studies, such as this study. A small-to-medium effect size in combination with an R-squared value between 0 and 1 suggests that this study might have limited power. For future confirmatory studies, a larger sample size representing the Saudi population would gather more information on the detrimental effects of driving stress.

## 5. Conclusions

Driving stress-induced driving behaviors significantly resulted in pain in the facial region, the audibility of TMJ sounds, and limitations in the opening and closure of the oral cavity. A dislike of driving and annoyance when being overtaken were associated with the orofacial structure and functioning and had a significant influence on pain in mastication and chewing. Many parafunctional habits positively correlated with driving aggression and driving dislikes. This study highlights the deleterious impacts of driving stress on orofacial functions and health behaviors. The short-term stress response effects during driving stress help in adaptive responses. However, long-term driving stress can be maladaptive and can increase the risk of chronic temporomandibular disorders; changes in the orofacial structures and hard tissue structures of the teeth due to parafunctional habits; and detrimental effects of health-behavior habits such as smoking, an excessive consumption of beverages, and excessive sitting while driving, with all of these leading to the risk of chronic cardiovascular and metabolic diseases.

## Figures and Tables

**Table 1:** Demographic data.

Parameters	Total Subjects (N = 161)	
	N	%
**Age (%)**		
18–28	90	55.9
29–38	54	33.5
39–48	14	8.7
49–58	3	1.9
**Gender (%)**		
Male	86	53.4
Female	75	46.6
**Marital status (%)**		
Single	115	71.4
Married	46	28.6
**Level of Education (%)**		
Below Grade 10	7	4.3
Up to Grade 10	2	1.2
High School/Diploma	42	26.1
Bachelor’s Degree	83	51.6
Postgraduate Degree	23	14.3
PHD	4	2.5
**Employment Status (%)**		
Student	48	29.8
Unemployed	7	4.3
Employed	105	65.2
**Place Of residence in Riyadh (%)**		
City Centre	103	64.0
Outskirts of the city	58	36.0
**Hours Of day spent in driving (%)**		
Less than 30 min	9	5.6
30 min to 1 h	31	19.3
1 h to 2 h	42	26.1
More than 2 h	79	49.1

**Table 2:** Profile of habitual information of the subjects.

Habitual Information	Total Subjects (N = 161)	
	N	%
**Smoking while driving (%)**		
Yes	48	29.8
No	113	70.2
**Consumption of beverages while driving (%)**		
Yes	111	68.9
No	50	31.1
**Type of beverages consumed (%)**		
Tea	19	11.8
Coffee	87	54.0
Energy drinks	4	2.5
Juice	2	1.2
Soft Drinks	4	2.5
I do not consume beverages. Only water	31	19.3
No beverages. No water	14	8.7
**Excessive sitting hours (%)**		
Less than 8 h	56	34.8
More than 8 h	105	65.2
**Insufficient Physical activity (%)**		
Less than 150 min/week	105	65.2
More than 150 min/week	56	34.8
**Insufficient sleep (%)**		
Less than 7 h/day	107	66.5
More than 7 h/day	54	33.5
**Time Stress (%)**		
Never	21	13.0
Rarely	12	7.5
Sometimes	34	21.1
Often	36	22.4
Always	58	36.0
**Health interfering with social activities (%)**		
Not at all	45	28.0
Less often	30	18.6
Most of the time	56	34.8
All of the time	30	18.6

**Table 3:** Correlation between driving stress and effects on orofacial structures.

Parameters	R-Value	Effect Size	*p* Value
Nail biting vs. Driving aggression	0.16	0.25	* 0.04
Nail biting vs. Dislike of driving	0.07	0.21	0.39
Nail biting vs. Tension and frustration linked overtaking	0.15	0.21	0.06
Nail biting vs. Annoyance when being overtaken	0.13	0.18	0.09
Nail biting vs. Heightened alertness and concentration	0.07	0.19	0.37
Grinding of teeth vs. Driving aggression	−0.02	0.19	0.81
Grinding of teeth vs. Dislike of driving	0.20	0.19	* 0.01
Grinding of teeth vs. Tension and frustration linked overtaking	0.14	0.22	0.07
Grinding of teeth vs. Annoyance when being overtaken	0.12	0.23	0.14
Grinding of teeth vs. Heightened alertness and concentration	−0.12	0.21	0.15
Clenching of the Jaws vs. Driving aggression	0.00	0.21	0.96
Clenching of the Jaws vs. Dislike of driving	0.16	0.19	* 0.04
Clenching of the Jaws vs. Tension and frustration linked overtaking	0.14	0.22	0.09
Clenching of the Jaws vs. Annoyance when being overtaken	0.11	0.21	0.16
Clenching of the Jaws vs. Heightened alertness and concentration	0.00	0.15	0.97
Lip or object biting vs. Driving aggression	0.17	0.20	* 0.03
Lip or object biting vs. Dislike of driving	0.19	0.20	* 0.02
Lip or object biting vs. Tension and frustration linked overtaking	0.15	0.19	0.06
Lip or object biting vs. Annoyance when being overtaken	0.13	0.16	0.10
Lip or object biting vs. Heightened alertness and concentration	−0.02	0.20	0.84
Chewing gums vs. Driving aggression	0.06	0.22	0.47
Chewing gums vs. Dislike of driving	0.03	0.15	0.71
Chewing gums vs. Tension and frustration linked overtaking	0.01	0.20	0.94
Chewing gums vs. Annoyance when being overtaken	−0.05	0.16	0.50
Chewing gums vs. Heightened alertness and concentration	0.16	0.18	* 0.04
Pain in facial region vs. Driving aggression	0.02	0.24	0.83
Pain in facial region vs. Dislike of driving	0.02	0.18	0.77
Pain in facial region vs. Tension and frustration linked overtaking	0.13	0.17	0.11
Pain in facial region vs. Annoyance when being overtaken	0.25	0.35	* 0.00
Pain in facial region vs. Heightened alertness and concentration	0.12	0.13	0.13
Any episodes of headache vs. Driving aggression	0.03	0.16	0.68
Any episodes of headache vs. Dislike of driving	0.05	0.22	0.51
Any episodes of headache vs. Tension and frustration linked overtaking	0.11	0.21	0.18
Any episodes of headache vs. Annoyance when being overtaken	0.14	0.21	0.07
Any episodes of headache vs. Heightened alertness and concentration	0.10	0.21	0.23
Audibility of TMJ sounds vs. Driving aggression	0.06	0.24	0.42
Audibility of TMJ sounds vs. Dislike of driving	0.09	0.15	0.25
Audibility of TMJ sounds vs. Tension and frustration linked overtaking	0.10	0.12	0.22
Audibility of TMJ sounds vs. Annoyance when being overtaken	0.24	0.29	* 0.00
Audibility of TMJ sounds vs. Heightened alertness and concentration	0.08	0.11	0.29
Limitation in opening of the oral cavity and closure vs. Driving aggression	0.06	0.19	0.49
Limitation in opening of the oral cavity and closure vs. Dislike of driving	0.07	0.08	0.38
Limitation in opening of the oral cavity and closure vs. Tension and frustration linked overtaking	0.09	0.21	0.27
Limitation in opening of the oral cavity and closure vs. Annoyance when being overtaken	0.16	0.25	* 0.04
Limitation in opening of the oral cavity and closure vs. Heightened alertness and concentration	0.08	0.2	0.34
Pain on mastication vs. Driving aggression	0.06	0.22	0.45
Pain on mastication vs. Dislike of driving	0.17	0.19	* 0.04
Pain on mastication vs. Tension and frustration linked overtaking	0.14	0.20	0.09
Pain on mastication vs. Annoyance when being overtaken	0.24	0.33	* 0.00
Pain on mastication vs. Heightened alertness and concentration	0.14	0.17	0.08
Tiredness in muscles vs. Driving aggression	−0.11	0.17	0.18
Tiredness in muscles vs. Dislike of driving	0.03	0.11	0.70
Tiredness in muscles vs. Tension and frustration linked overtaking	0.07	0.07	0.39
Tiredness in muscles vs. Annoyance when being overtaken	0.04	0.12	0.58
Tiredness in muscles vs. Heightened alertness and concentration	0.07	0.14	0.40

Pearson’s correlation co-efficient test, *p* < 0.05, * significant.

**Table 4:** Results of regression analysis that evaluated the association between driving behaviors and their effects on the orofacial region and functions.

Parameters	β-Value	Effect Size	95% CI	* R^2^	*p* Value
Pain in facial region vs. Driving aggression	1.03	0.02	0.81–1.31	0.0003	0.833
Pain in facial region vs. Dislike of driving	1.04	0.02	0.81–1.34	0.0005	0.767
Pain in facial region vs. Tension and frustration linked overtaking	1.21	0.13	0.96–1.52	0.016	0.111
Pain in facial region vs. Annoyance when being overtaken	1.46	0.25	1.15–1.86	0.0636	* 0.002
Pain in facial region vs. Heightened alertness and concentration	1.34	0.12	0.94–1.60	0.0141	0.134
Any episodes of headache vs. Driving aggression	1.05	0.03	0.83–1.32	0.0011	0.667
Any episodes of headache vs. Dislike of driving	1.09	0.05	0.85–1.39	0.0027	0.507
Any episodes of headache vs. Tension and frustration linked overtaking	1.16	0.11	0.93–1.45	0.0115	0.175
Any episodes of headache vs. Annoyance when being overtaken	1.22	0.14	0.98–1.52	0.0201	0.075
Any episodes of headache vs. Heightened alertness and concentration	1.16	0.10	0.91–1.48	0.0091	0.227
Audibility of TMJ sounds vs. Driving aggression	1.10	0.06	0.87–1.40	0.0041	0.418
Audibility of TMJ sounds vs. Dislike of driving	1.15	0.09	0.90–1.48	0.0082	0.253
Audibility of TMJ sounds vs. Tension and frustration linked overtaking	1.15	0.10	0.92–1.44	0.0095	0.218
Audibility of TMJ sounds vs. Annoyance when being overtaken	1.43	0.24	1.13–1.81	0.0582	* 0.003
Audibility of TMJ sounds vs. Heightened alertness and concentration	1.15	0.08	0.89–1.49	0.0071	0.288
Limitation in opening of the oral cavity and closure vs. Driving aggression	1.09	0.06	0.85–1.41	0.0031	0.482
Limitation in opening of the oral cavity and closure vs. Dislike of driving	1.13	0.07	0.87–1.46	0.0049	0.378
Limitation in opening of the oral cavity and closure vs. Tension and frustration linked overtaking	1.14	0.09	0.90–1.46	0.0075	0.272
Limitation in opening of the oral cavity and closure vs. Annoyance when being overtaken	1.28	0.16	1.00–1.63	0.0246	* 0.049
Limitation in opening of the oral cavity and closure vs. Heightened alertness and concentration	1.15	0.08	0.87–1.51	0.0058	0.335
Pain in mastication vs. Driving aggression	1.11	0.06	0.85–1.45	0.0037	0.442
Pain in mastication vs. Dislike of driving	1.33	0.17	1.02–1.75	0.0275	* 0.039
Pain in mastication vs. Tension and frustration linked overtaking	1.25	0.13	0.97–1.62	0.0181	0.091
Pain in mastication vs. Annoyance when being overtaken	1.52	0.24	1.15–1.99	0.0594	* 0.003
Pain in mastication vs. Heightened alertness and concentration	1.31	0.14	0.96–1.79	0.0186	0.087
Tiredness in muscles vs. Driving aggression	0.86	0.11	0.69–1.07	0.0112	0.181
Tiredness in muscles vs. Dislike of driving	1.05	0.03	0.83–1.32	0.0009	0.700
Tiredness in muscles vs. Tension and frustration linked overtaking	1.10	0.07	0.89–1.35	0.0047	0.384
Tiredness in muscles vs. Annoyance when being overtaken	1.06	0.04	0.86–1.30	0.0019	0.576
Tiredness in muscles vs. Heightened alertness and concentration	1.11	0.07	0.88–1.40	0.0046	0.393

* R-squared value indicates the goodness of fit of the data to the regression model and the power.

## Data Availability

The data are available upon request from the corresponding authors.
